# Recent advances in understanding and managing cutaneous T-cell lymphomas

**DOI:** 10.12688/f1000research.21922.1

**Published:** 2020-05-05

**Authors:** Patrick M. Brunner, Constanze Jonak, Robert Knobler

**Affiliations:** 1 Department of Dermatology, Medical University of Vienna, Währinger Gürtel 18-20, Vienna, 1090, Austria

**Keywords:** Cutaneous T cell lymphoma, Mycosis fungoides, Sézary Syndrome, health-related quality of life, tissue resident memory T cells

## Abstract

Cutaneous T-cell lymphomas (CTCLs) comprise a heterogeneous group of extranodal non-Hodgkin lymphomas involving primarily the skin and mycosis fungoides is its most frequent entity. Whereas most patients show an indolent course in early disease (clinical stages IA to IIA), some patients progress to advanced disease (stage IIB or higher), and the 5-year survival rate is unfavorable: only 47% (stage IIB) to 18% (stage IVB). Except for allogeneic stem cell transplantation, there is currently no cure for CTCL and thus treatment approaches are palliative, focusing on patients’ health-related quality of life. Our aims were to review the current understanding of the pathogenesis of CTCL, such as the shift in overall immune skewing with progressive disease and the challenges of making a timely diagnosis in early-stage disease because of the lack of reliable positive markers for routine diagnostics, and to discuss established and potential treatment modalities such as immunotherapy and novel targeted therapeutics.

## Introduction

Cutaneous T-cell lymphomas (CTCLs) are primary lymphomas of the skin, and the estimated incidence is about 5.6 per million ^1, 2^. The term CTCL comprises a group of malignancies that develop from skin-homing T cells, which are of the CD4 ^+^ T helper cell type in more than 90% of cases ^1, 2^. The most frequent clinical entity of CTCL is mycosis fungoides (MF), accounting for about 60% of all cases, followed by primary cutaneous CD30 ^+^ lymphoproliferative disorders and much rarer entities such as Sézary syndrome (SS). The 2018 update of the World Health Organization–European Organization for Research and Treatment of Cancer (WHO-EORTC) classification for cutaneous lymphomas describes new provisional entities—namely chronic active Epstein–Barr virus infection, CD8 ^+^ aggressive epidermotropic CTCL, primary cutaneous CD4 ^+^ small/medium T-cell lymphoproliferative disorder, and primary acral CD8 ^+^ T-cell lymphoma—which are now included ^3^. In early disease stages, MF presents clinically with erythematous patches or plaques and this stage can last for many years without clinical progression and without affecting the life expectancy of the patient ^4^. Nevertheless, CTCLs may be disfiguring and thus have an impact on patients’ health-related quality of life (HRQoL) ^5, 6^. In about 30% of patients presenting with early-stage CTCL, the disease progresses to advanced-stage disease within 10 years ^7^ by gradually developing into tumors and may disseminate to lymph nodes, blood, and internal organs, resulting in an unfavorable prognosis ^8, 9^; 5-year overall survival rates decrease from 94% in stage IA to as low as 18% in stage IVB ^10^. However, in rare cases, MF can also initially present with tumors representing the disease variant “tumour d’emblée”. It is still unclear why about 30% of patients with CTCL ^7^ progress to advanced disease; currently, prediction of prognosis is based predominantly on clinical staging by using the TNMB (tumor, node, metastasis, blood) classification for MF/SS ^11^ and there are additional roles for gender, age, blood lactate dehydrogenase concentration, folliculotropism, large-cell transformation, and the detection of clonality ^10^. Of note, no validated biomarkers, favorable or unfavorable, are available for the prediction of the disease course ^12^.

## Disease etiology and pathogenesis

The etiology of most variants of CTCL remains only poorly understood. Potential etiologic factors include infectious agents, ultraviolet (UV) light, or occupational exposures ^13, 14^. Large-scale mutational genome profiling analyses identified genomic alterations in several putative oncogenes and tumor suppressor genes, including *CARD11*, *CCR4, TP53, NF-κB*, and Janus kinase signaling members ^15– 19^, but heterogeneity between individual patients can be considerable ^15– 19^. It is assumed that, besides a dysfunctional regulation of cytokines and other signaling molecules that most likely play a decisive role for malignant transformation, epigenetic modifications such as pathologic gene methylation and histone deacetylation play a role in malignant transformation of CTCL cells ^20^.

In MF, the malignant T cells have been identified as typically CD4 ^+^ skin-resident effector memory T cells of clonal origin ^21^. Their skin-resident nature explains why MF lesions tend to be primarily present in the skin in most patients ^21, 22^. By contrast, SS cells are classified as of the central memory phenotype (expressing the lymph node homing molecules CCR7 and L-selectin), enabling these cells to freely move from the periphery to lymphoid organs ^21, 22^. Consistently, the phenotype of SS typically shows erythroderma as well as blood and lymph node involvement.

CTCL cells have been described to express cytokines of various helper cell subtypes, including T helper 2 (Th2), Th17, and regulatory T cells ^23– 25^. In addition, many other chemokines and cytokines are being produced within lesions, adding to the overall complexity of the disease ^26^. Histologically, malignant T cells are characterized by epidermotropism—that is, they are preferentially present in the upper parts of the skin (epidermis)—whereas non-malignant T cells are detected primarily in the dermis, especially in early MF. This early immune infiltrate consists primarily of non-malignant Th1 cells, regulatory T cells, and cytotoxic CD8 ^+^ T cells (tumor-infiltrating lymphocytes, or TILs), which are hypothesized to (initially) keep the malignant T cells under control ^27, 28^, but the precise mechanisms remain unclear. During progression of the disease, a switch from the benign “bystander” infiltrate of Th1 cells and CD8 ^+^ TILs to a more Th2-biased phenotype, including the appearance of blood eosinophilia and markedly elevated IgE blood levels ^26^, has been documented ^29^. Conversely, therapeutic success in MF during skin-directed psoralen plus UVA (PUVA) therapy was linked to a shift from Th2 toward a Th1 phenotype with clearance of the skin lesions irrespective of tumor cell burden, implicating an increased turnover of benign T cells ^30^. Thus, Th1 immune skewing in early-stage disease is traditionally considered protective (produced by the accompanying “anti-tumor” infiltrate), whereas a gradual loss of this Th1 signature, with an increase in Th2 mediators, is regarded as a sign of disease progression ^31^. It is assumed that, mechanistically, tumor-derived Th2 cytokines suppress proliferation of benign T cells and inhibit dendritic cell (DC) maturation ^32^. Such a Th2-dominant skin immune skewing is also found in atopic dermatitis; intriguingly, both patients with MF and those with atopic dermatitis have *Staphylococcus aureus* colonization and increased rates of infectious complications ^33, 34^. Recent data even suggest that staphylococcal alpha toxin itself may promote disease progression through positive selection of CD4 ^+^ tumor cells ^35^. In line with its opposing effect toward Th2-associated inflammation, the major Th1 cytokine interferon-gamma (IFN-γ) shows some efficacy in CTCL treatment ^36^. However, other cells, including fibroblasts, keratinocytes, and endothelial cells, are thought to promote and augment a Th2 microenvironment in advanced-stage MF, thereby further attenuating Th1 immune responses ^37^.

DCs have the unique capacity to induce primary immune responses by activating naïve T cells and thus are the central gatekeepers for the initiation of adaptive immune responses ^38^. As recently shown, c-Kit ^+^OX40L ^+^CD40L ^+^ DCs can foster the visible skin inflammation within skin lesions by recruiting and activating benign T cells and this mechanism likely provides key tumorigenic signals within the CTCL immune microenvironment ^30^. In advanced-stage CTCL, the maturation of DCs is thought to be suppressed by Th2 cytokines ^32^. Importantly, immature DCs can induce tolerance by presenting antigens to T cells without appropriate co-stimulation, thus fostering a tumor-tolerating (micro)environment rather than an anti-tumor immune defense ^39^. Consistently, increased levels of immature DCs are found in MF lesions, which might be an important mechanism for tolerance against malignant T cells ^40^.

Keratinocytes produce multiple chemokines, including CCL17, CCL26, CCL27, CXCL9, and CXCL10, which are potent chemo-attractants for several immune cell populations. They also produce nerve growth factor, which is suggested to be involved in itch development, a typical symptom for CTCL ^26^. Mast cells might also be involved in CTCL pathogenesis, as their number correlates with disease progression ^41^. Similarly, myeloid-derived suppressor cells increase with advanced disease stage ^42^, and immunosuppressive M2 macrophages are known to promote tumor growth in various cancers ^43^ and could also play a role in CTCL ^44– 46^. Overall, there is a complex interplay between tumor cells and the tissue microenvironment, which is not yet fully elucidated.

## Diagnosis of disease

In CTCL, genetic markers are currently under intense investigation as potential diagnostic tools, but single diagnostic biomarkers are still lacking. Thus, the integration of clinical morphology, histology, immune-phenotype, and molecular biological data remains essential for an accurate diagnosis ^3^. Accordingly, the diagnosis of CTCL is based on the combination and correlation of the three following assessments: (a) clinical observations, (b) (immuno)histological examination of skin biopsies, and (c) additional laboratory tests such as flow cytometry of peripheral blood and the analysis of T-cell receptor (TCR) clonality by polymerase chain reaction (PCR) ^47^. The parameter of large-cell transformation of MF cells, based on histological criteria, is found in 56 to 67% of patients with advanced-stage MF ^9^ and linked to an aggressive disease course with shortened survival. Besides malignant T cells, an abundant number of reactive immune cells, including high numbers of non-malignant T cells, accompany the malignant clone. Molecular and immunohistochemistry markers that are currently used to diagnose MF are usually negative markers, such as loss of expression of, for example, CD7 or CD26, but this kind of aberrant surface expression shows considerable variability from case to case ^48^. Useful positive diagnostic markers are still lacking for routine diagnostics. Importantly, the actual lymphoma cells are present in only small numbers during early stages of the disease. Thus, analyses of clonality (TCR rearrangement) are often falsely negative in early MF ^49^. Rea *et al.* showed that, for CTCL diagnosis, high-throughput sequencing was more specific than *TCR gamma chain* gene PCR (100% versus 88%) but that sensitivity (68% versus 72%) and accuracy (84% versus 80%) were similar ^50^. Thus, high-throughput sequencing, assessing both clonality and T-cell fractions in skin biopsies, is a promising tool to increase diagnostic accuracy in CTCL ^50^.

TOX (thymocyte selection-associated high-mobility group box) has been found to be strongly upregulated in early MF compared with lower levels in benign inflammatory dermatitis ^51^. Although TOX has insufficient sensitivity and specificity to serve as a single diagnostic marker, it might have an adjunctive diagnostic role together with other clinical and histological data ^52^. For SS, CD27 might serve as a diagnostic tool to distinguish this disease from benign inflammatory erythroderma ^53^. Nevertheless, the overall lack of validated and specific diagnostic markers adds to the fact that, in MF, for instance, it takes a median of about 3 years ^7^ from the initial appearance of skin lesions until a definitive diagnosis can be made, demonstrating the difficulty of making a timely diagnosis, which mandates further research.

## Current lack of prognostic biomarkers that can predict disease progression

The prediction of disease progression and overall survival in patients with MF has turned out to be a challenging endeavor ^54^. A Cutaneous Lymphoma International Prognostic Index (CLIPI), which includes age, gender, folliculotropism, and TNMB staging, has been developed but has limited utility in early-stage patients ^55, 56^. The CXCR4–CXCL12 axis has been described as a potential player in MF progression ^57^, but other authors did not consistently find differences between early and late disease stages ^58^. Recently, however, De Masson *et al.* used high-throughput sequencing of the *TCR beta* gene and found that an increased frequency of the malignant T-cell clone in MF skin lesions is strongly correlated with reduced overall survival in patients with early-stage disease ^55^.

Several authors showed associations of poor disease outcome with *TOX* ^59, 60^ as well as *miR-155, miR-21*, and *let-7i* microRNA expression ^17, 47^. Lefrançois *et al.* ^54^ described *TOX*, *FYB, CCR4*, and *CD52* as markers for disease progression and decreased survival in MF, and the authors confirmed these markers in an independent cohort of patients with SS. KIR3DL2, a recently discovered marker of the malignant clonal cell population in SS, was suggested as an independent prognostic factor for SS-specific death ^61^. Nevertheless, none of these potential biomarkers has been fully validated so far ^55, 62^ and thus they are not yet available for clinical use.

One major factor limiting our insight into MF/SS pathogenesis is likely rooted in the considerable disease heterogeneity seen between individual patients ^63^. Importantly, significant heterogeneity of gene expression was present not only between different patients but also within the same individual over time ^12, 64, 65^. Importantly, all of these studies used bulk tissue investigations or conventional immunohistochemistry studies or both. However, bulk tissue investigations do not distinguish between individual cell types (for example, the actual tumor cell and the tumor microenvironment), and conventional measurements of cell heterogeneity such as immunohistochemistry can suffer from artifacts and are impractical when several markers are investigated at the same time ^66^. Single-cell RNA sequencing has been shown to be a powerful tool to characterize complex as well as rare immune cell populations and to reveal unexpected or undescribed cell subpopulations, allowing the acquisition of large amounts of transcriptional information for the accurate and unbiased molecular characterization of these rare cells ^67^. Gaydosik *et al.* ^63^ performed a first investigation in advanced-stage CTCL and healthy control samples using single-cell RNA sequencing and found large inter- and intra-tumor gene expression heterogeneity in the T lymphocyte subset. Borcherding *et al.* ^68^ investigated CTCL heterogeneity in circulating SS cells, giving insight into the heterogeneity of SS cells within a single patient and putting transcriptomic differences into context with published MF data. Though still at the beginning in CTCL, the evolution of single-cell transcriptomics might offer exciting new opportunities to better understand tumor cell heterogeneity and to facilitate the development of stratified diagnostic and prognostic approaches for patients with CTCL.

## Novel treatment approaches

There is currently no cure for CTCL, except for allogeneic stem cell transplantation, which carries a significant risk of relapse as well as treatment-related toxicity and death ^69^. Immunotherapy including checkpoint inhibitors for CTCL is only at the beginning and might be a promising tool to achieve long-term disease control ^70^, but the results of ongoing clinical trials must be awaited before making firm conclusions. Currently, CTCL treatment is based on a stage-related therapeutic approach. Thus, early-stage disease is first treated with skin-directed therapies ^2, 71^. These include topical application of glucocorticosteroids, topical mechlorethamine ^72^, topical bexarotene, and the use of phototherapy (UVB and PUVA) ^73^ or localized radiation therapy (photon or electron beam). In addition, early-phase clinical trials or case series (or both) suggest a potential role for topical Toll-like receptor (TLR) agonists such as imiquimod ^74^ or resiquimod ^75^, photodynamic therapy ^76^, and excimer laser therapy ^77^, but data are limited and need corroboration in larger clinical trials. Interestingly, resiquimod showed regression not only of treated but also of untreated lesions in a phase 1 study ^75^ and is being further investigated as a skin-directed compound.

Widespread, advanced disease or refractory early-disease requires systemic therapy. Established treatment modalities include low-dose methotrexate, systemic bexarotene, IFN, the histone deacetylase inhibitors romidepsin and vorinostat, the anti-CD52 antibody alemtuzumab ^78^, extracorporeal photopheresis, and single-agent or combination chemotherapy.

Mogamulizumab is a monoclonal antibody directed against the chemokine receptor CCR4 and has been investigated in a phase 3 trial in comparison with vorinostat in patients with previously treated CTCL ^79^. The investigators showed that mogamulizumab resulted in a median progression-free survival of 7.7 months, compared with only 3.1 with the active comparator, and an objective response rate of 28% versus 5%. Surprisingly, vorinostat response rates were actually lower than in initial studies ^80, 81^. Response rates to mogamulizumab were independent of previous therapies ^82^; long-term treatment seems to be safe and well tolerated ^82^. Thus, mogamulizumab might also be an option for heavily pre-treated patients, and besides MF, this agent seems to be particularly promising for patients with SS ^79^.

Brentuximab vedotin is an antibody–drug conjugate designed to target CD30 in CD30 ^+^ CTCL ^83^. Although efficacy was reported to be better in CD30-expressing CTCL, clinical responses were still observed in patients with lower CD30 expression ^82^. Thus, this treatment might also be an option for a wider range of CTCLs beyond those highly expressing CD30.

Owing to the chronic-relapsing nature of CTCLs, there are also investigations into maintenance therapy using lower treatment doses or frequencies or both. There are some promising data on maintenance PUVA therapy extending median disease-free remission from 4 to 15 months ^73^. Resminostat is being investigated in a phase 2 clinical trial for maintenance therapy in patients with advanced-stage MF or SS (ClinicalTrials.gov Identifier: NCT02953301). Another promising treatment modality includes low-dose total skin electron beam treatment followed by mechlorethamine ^84^, which is being investigated in a phase 2 trial (ClinicalTrials.gov Identifier: NCT02881749). A phase 3 trial on lenalidomide maintenance in advanced CTCL was terminated early following withdrawal of funding support and thus was underpowered for analysis ^85^.

Immune checkpoint inhibitors have revolutionized treatment of many cancer types, and there have been durable responses in some patients even after cessation of therapy ^86^. As briefly mentioned above, these new agents might also be an option for CTCL ^87^. In a phase II trial of patients with advanced, heavily pre-treated MF and SS, the PD-1 inhibitor pembrolizumab demonstrated significant anti-tumor activity and had overall response rates of 38% and a favorable safety profile ^88^. Ongoing treatment responses at last follow-up were observed in one third of patients ^88^. Besides pembrolizumab, the PD-1 blocker nivolumab and the PD-L1 inhibitors durvalumab and atezolizumab are being investigated in clinical trials. Despite promising initial results, caution needs to be exerted as PD-1 blockade might also accelerate growth of T-cell malignancies, as seen in adult T-cell leukemia/lymphoma that showed explosive progression of disease in the first three patients ^89^.

Other treatment approaches being evaluated in early clinical trials include the SYK/JAK inhibitor cerdulatinib, an anti-KIR antibody (IPH4102), the microRNA Mir-155 inhibitor cobomarsen, and the CD70 blocker cusatuzumab ^82^.

## Outlook

Our understanding of the pathogenesis of CTCL has steadily increased within the last few years, but many aspects are still only insufficiently understood, necessitating further basic and translational research approaches. Whereas many patients show an indolent and self-limiting course, some patients progress. For those patients, treatment options are often limited and can harbor significant toxicity. In line with this, early-stage disease should preferentially be treated with skin-directed therapies and, in case of relapse and after initial therapeutic success, should be used in a repetitive manner. Systemic treatment should preferably be kept for advanced-stage disease or patients with refractory cutaneous involvement (for example, after secondary loss of efficacy with skin-directed therapies). Generally, the management of patients with CTCL should be reserved for specialized centers, which also offer the advantage of access to multicenter clinical trials. It is noteworthy that HRQoL is of significant importance in CTCL, profoundly influencing patients’ quality of life in terms of a visible stigma and its potential lethality ^90^. Accordingly, HRQoL and its maintenance in MF/SS are of utmost importance in the face of current palliative treatment approaches in the majority of cases.
